# Heart Metabolism in Sepsis-Induced Cardiomyopathy—Unusual Metabolic Dysfunction of the Heart

**DOI:** 10.3390/ijerph18147598

**Published:** 2021-07-16

**Authors:** Weronika Wasyluk, Patrycja Nowicka-Stążka, Agnieszka Zwolak

**Affiliations:** 1 Chair of Internal Medicine and Department of Internal Medicine in Nursing, Faculty of Health Sciences, Medical University of Lublin, 20-093 Lublin, Poland; patrycjanowickastazka@umlub.pl (P.N.-S.); agnieszka.zwolak@umlub.pl (A.Z.); 2 Doctoral School, Medical University of Lublin, 20-093 Lublin, Poland

**Keywords:** heart failure, sepsis, sepsis-induced cardiomyopathy, cardiac metabolism, metabolic remodelling, intensive care

## Abstract

Due to the need for continuous work, the heart uses up to 8% of the total energy expenditure. Due to the relatively low adenosine triphosphate (ATP) storage capacity, the heart’s work is dependent on its production. This is possible due to the metabolic flexibility of the heart, which allows it to use numerous substrates as a source of energy. Under normal conditions, a healthy heart obtains approximately 95% of its ATP by oxidative phosphorylation in the mitochondria. The primary source of energy is fatty acid oxidation, the rest of the energy comes from the oxidation of pyruvate. A failed heart is characterised by a disturbance in these proportions, with the contribution of individual components as a source of energy depending on the aetiology and stage of heart failure. A unique form of cardiac dysfunction is sepsis-induced cardiomyopathy, characterised by a significant reduction in energy production and impairment of cardiac oxidation of both fatty acids and glucose. Metabolic disorders appear to contribute to the pathogenesis of cardiac dysfunction and therefore are a promising target for future therapies. However, as many aspects of the metabolism of the failing heart remain unexplained, this issue requires further research.

## 1. Introduction

Despite advances in knowledge and medicine and a significant reduction in mortality, heart failure remains a significant challenge [1]. There are many methods of treatment, mainly focusing on haemodynamic and neurohormonal factors, but in some patients, the results of treatment remain unsatisfactory. This prompts the search for alternative treatment strategies [2]. A promising area for this research is the issue of myocardial metabolism. It has been shown that heart failure is accompanied by numerous metabolic disorders of this organ, which is particularly important due to the continuous contractile activity and relatively high energy demand.

The state which requires the effect of such searches is sepsis-induced cardiomyopathy (SICM). Sepsis has been described as ‘life-threatening organ dysfunction caused by a dysregulated host response to infection’ and is considered the most common cause of death in critically ill patients [3,4,5]. One of the organs most frequently affected by sepsis dysfunction is the heart [6]. Despite numerous similarities, metabolic disorders in the course of SICM are characteristic; therefore, their discussion requires both an understanding of the disorders occurring in the failing heart and highlighting the disorders typical of sepsis.

A better understanding of the metabolic aspect of sepsis-induced cardiomyopathy could reveal new therapeutic targets. The purpose of this paper is to summarise the metabolic changes occurring in heart failure and then to present reports on disorders of heart metabolism in SICM.

## 2. Physiology of Heart Metabolism

The energy demand of the heart muscle is very high, and its work requires the constant availability of the fuel, which is adenosine triphosphate (ATP) [2,7]. Despite the relatively small size of the heart (about 0.5% of body weight), ATP consumed by it accounts for up to 8% of the entire body’s energy requirements [8]. Due to the relatively low ATP storage capacity in the myocardium, the heart’s work is closely dependent on its continuous production [2,7]. Most of the energy consumed by the heart is absorbed by processes of excitation–contraction coupling, and the main consumers of ATP are myosin ATPase, sarcoendoplasmic reticulum Ca^2+^-ATPase (SERCA), and the Na^+^/K^+^-ATPase [9,10,11]. The production of ATP occurs through the catabolism of a number of substrates, including fatty acids (FAs), glucose, ketone bodies (KB), and amino acids (AA) [12]. Due to the ability to use various substrates, the heart is referred to in the literature as a ‘metabolic omnivore’ [2,9,12,13]. The key feature of the heart, which allows it to use a variety of substrates, is metabolic flexibility—it enables a change in the type and quantity ratio of the substrates used in response to changes in workload, substrate availability, and the hormonal environment [9,12].

**Figure 1:**
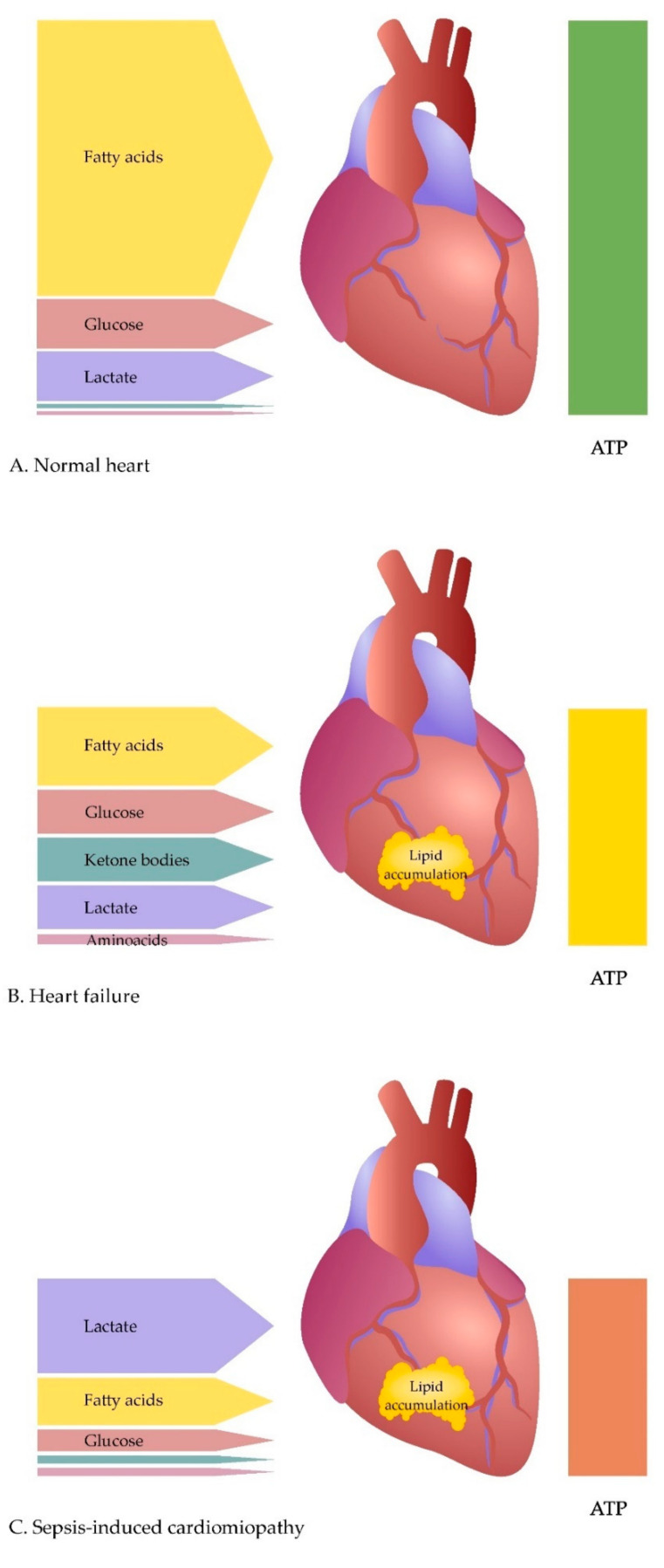
Comparison of energy metabolism in a healthy heart (**A**), in the course of heart failure (**B**), and sepsis-induced cardiomyopathy (**C**). Abbreviations: ATP = adenosine triphosphate.

Under normal conditions, a healthy heart obtains approximately 95% of its ATP by oxidative phosphorylation in the mitochondria. The primary source of energy is mitochondrial fatty acid oxidation (FAO), which is the source of approximately 60–90% of ATP. The rest of the energy (10–40% ATP) comes from the oxidation of pyruvate, which is produced by glycolysis and lactate oxidation. The remaining substrates, including KB and AA, under physiological conditions, constitute minor additional sources of energy (Figure 1A) [9,14].

The oxidation of energy substrates in the heart depends primarily on the catalytic activity of enzymes and the number of their molecules in the cells. Catalytic activity can be regulated by post-translational modification of enzymes (phosphorylation, acetylation) and their binding to proteins and allosteric effectors, while the number of enzyme molecules is a result of the processes of transcription and translation [9]. The phenomenon of mutual competitive regulation of glucose and FA oxidation, called the Randle cycle, is also known. Randle et al. have shown that the use of one of these nutrients inhibits the use of the other. Acetyl coenzyme A (acetyl–CoA), a product of β-oxidation of FAs, inhibits the activity of pyruvate dehydrogenase (PDH), and citrate (the product of the first tricarboxylic acid (TCA) cycle reaction after acetyl–CoA activation) inhibits phosphofructokinase, a key enzyme in the process of glycolysis [15].

Transcription of genes encoding enzymes related to oxidation and transport of energy substrates is regulated by transcription factors. These include the peroxisome proliferator-activated receptor α (PPARα), a receptor from the nuclear receptor family that regulates the expression of genes encoding proteins involved in the FA β-oxidation pathway. In turn, the activation of PPARα requires the participation of a peroxisome proliferator-activated receptor γ coactivator 1-α (PGC-1α) or PGC-1β [4,12]. PGC-1α also interacts with the oestrogen-like receptor α (ERRα) and regulates the biogenesis of mitochondria, the transport of FAs and glucose to the mitochondria, and the synthesis of ATP [16,17,18]. Moreover, activated PGC-1α coactivates nuclear receptor factors 1 and 2 (NRF1/2) and induces the transcription of mitochondrial genes [17,19,20]. The activation of NRF by PGC1α also induces mitochondrial transcription factor A (Tfam), which promotes the replication of mitochondria [19]. NRF1 has also been identified as a potential epigenetic-sensitive transcriptional regulator of cardiac metabolism and mitochondrial biogenesis [13,21,22].

Another factor regulating the metabolism of cardiomyocytes is the metabolic state of the cell itself. Research suggests that intermediates of metabolic processes can act as signalling agents, activating intracellular signalling cascades and inducing epigenetic and post-translational modifications [9]. The metabolism-related regulator is also 5’AMP-activated protein kinase (AMPK), an enzyme activated in response to an increase in the level of adenosine monophosphate (AMP), a marker of ATP depletion [23,24]. The activation of AMPK promotes CD36 and GLUT 4 translocation (for FA and glucose uptake, respectively), FA and glucose oxidation, and inhibits ATP consuming processes, e.g., biosynthesis [12,14,25,26].

## 3. Heart Metabolism in Heart Failure

There is a growing body of evidence pointing to the role of metabolic failure in the pathogenesis of heart failure (HF) [2,9,14,27,28]. It seems that the metabolic reactions occurring in the failing heart are primarily adaptive, with time becoming maladaptive, which plays a role in the pathogenesis of HF and leads to disease progression [29,30,31]. The metabolic factors contributing to the progression of HF include impaired utilisation of energy substrates (loss of metabolic flexibility) [12,14,32], energy deficit [33], and oxidative stress [9,34]. It has been shown that in a failing heart, compared to a healthy heart, there is a reduction in ATP production by up to 30–40%, a decrease in the creatine pool by 50–70%, and a reduction in the ratio of phosphocreatine content to ATP (Figure 1B) [32,35,36].

In HF, there is also a disturbance in the use of energy substrates—altered substrate preferences have been observed both in humans and in animal models [17]. Characteristic is a decrease in FAO share and an increase in glucose share in covering the energy requirements of cardiomyocytes [37,38]. The impaired oxidative metabolism of the heart in HF is compared to the metabolic profile of the foetal heart [12,13,29,39,40]. This metabolic reprogramming of the heart is mediated by the reactivation of genes responsible for glycolysis, with simultaneous suppression of genes involved in oxidative metabolism [13,41,42]. Moreover, patients with HF have been shown to overactivate the sympathetic nervous system, increase plasma catecholamines, and release angiotensin II. These factors may contribute to the reduction of oxidative metabolism, disturbing the oxidation of FAs and glucose [7,43,44]. In a failing heart, decreased PPARα and PGC-1α activity is observed, which is also considered to be an FAO reducing factor [16,17,45]. Moreover, the features of a failing heart include disturbances in mitochondrial function and impaired metabolic signalling [12,17].

It should be emphasised that considering the entire HF spectrum, the picture of metabolic disorders occurring in the heart is varied. Metabolic changes in cardiomyocytes depend on both the stage of HF and its aetiology [28,46]. Studies have shown that FAO in the early stages of HF may remain unchanged, and may even be slightly elevated [47,48,49], while glycolysis is increased and glucose uptake is increased [50]. Only in advanced or decompensated HF, the expression of enzymes participating in FAO is reduced and the role of FAs in ATP production is reduced [28,37,48,51,52]. In advanced HF, the efficiency of the glucose metabolism also decreases, and the share of KB as an energy substrate is increased [37,39,48]. The aetiology of HF influences the metabolic changes, for example, pressure loading or ischemia shifts metabolism to glucose utilisation, which does not occur with SICM [14,53,54,55]. In animal models, the rate of oxidation of different substrates varies which contributes to variability in research measurements [14,32].

### 3.1. Fatty Acids

As previously mentioned, FAs remain the main energy substrate of the heart under physiological conditions; however, mitochondrial FAO uses more oxygen per ATP molecule than most other energy substrates (Table 1). This makes FAs an inefficient energy source in terms of oxygen consumption [28,30]. In the failing heart, a metabolic shift from FAO to oxygen-sparing carbohydrate metabolism has been observed (glucose metabolism can provide 40% more ATP per oxygen molecule, compared to FA metabolism) [28,56,57]. Decreasing the use of FAs is one of the most common metabolic disorders in the failing heart and has been observed in both human and experimental models of HF [49,58,59,60]. It has been proposed that this shift is adaptive [12].

Although the FAO restriction is considered primarily adaptative, it may be part of the pathogenesis and cause of HF progression. Under physiological conditions, FAs delivered to the cell are activated—this reaction is irreversible and commits the FAs to the oxidation or resynthesis of triglycerides [63,64]. By limiting FAO, metabolic reprogramming causes a dyssynchronisation between substrate availability and its use [65]. This mismatch leads to intracellular lipid accumulation [56,66,67]. The excess of FAs partially accumulates in the myocardium in the form of triglycerides and partially is directed to non-oxidative pathways, leading to the formation of toxic lipid species (TLS) [68], which can damage mitochondria [69], modify cell signalling [70], and also enhance apoptosis (lipoapoptosis) [71,72]. Lipid accumulation may also lead to reaching levels that are harmful to cells by some of their metabolites, such as diacylglycerol and ceramide [9,68]. Increasing evidence also indicates the harmfulness of long-chain acylcarnitines (LCACs) accumulation, which can induce cellular stress and have pro-inflammatory and arrhythmogenic effects [64,73]. This phenomenon is referred to as lipotoxicity and, due to its detrimental effect on heart function, may contribute to the progression of HF [9,29,74,75].

### 3.2. Carbohydrates

In a failing heart, the share of glucose as an energy source may increase. It should be noted, however, that this increase is related to glucose uptake and glycolysis rate but not to glucose oxidation [9,52,76,77,78]. Moreover, studies show that even despite the relative increase in the share of glucose oxidation itself in energy production, the absolute flow of this substrate through the oxidative pathways decreases [9]. This may be related to a reduction in total mitochondrial oxidative metabolism [79,80], as well as a reduction in the availability of glucose due to impaired uptake by the heart in the course of possible insulin resistance occurring in HF (depending on its stage and form) [81,82]. In the context of the described limitations of the oxidative metabolism of carbohydrates, as well as the previously described reduction of FAO, the upregulation of glycolysis appears to be compensatory [77,79]. However, research results suggest that whether changes in cardiac glucose metabolism are adaptive or non-adaptive depends on the type and duration of HF [12]. In addition, the metabolic shift occurs in line with the previously discussed transition of the failing heart to the metabolic profile of the foetal heart.

### 3.3. Ketone Bodies

The heart, as a metabolic omnivore, easily uses KBs as a source of energy, and their oxidation is related to their availability [83]. Since KBs are normally not available in large amounts, their contribution to physiological cardiac energy metabolism is minimal [84,85]. Acetoacetate and β-hydroxybutyrate (βHB), the basic KBs involved in energy metabolism, are formed in the liver mitochondria during intensive oxidation of FA in the presence of their increased availability. This occurs, inter alia, in HF [86]. It has been shown that in HF, there is an increase in the concentration of KB in the blood and an increase in their metabolism in the myocardium [37,39,40,84,87,88]. In one of the key studies on this issue, Bedi et al. showed that the metabolic and genetic profile typical of the oxidation of KB was present only in failing hearts, which indicates that the shift to KB metabolism is a late event in the course of HF [37]. Based on these observations, it has been suggested that the metabolism of KB becomes more important in the failing heart when other pathways of other energy substrates become ineffective [37,89]. A study in a mouse HF model has shown that the oxidation of KB in the failing heart covers up to 27% of ATP production [88]. On the other hand, it should be noted that in some HF models, it has not been shown that HF is accompanied by an increased concentration of KB in the blood [39,90]. 

### 3.4. Amino Acids

Disturbances in the availability and metabolism of AAs have also been found in the course of HF. Although under physiological conditions, AAs in the heart are primarily a substrate in the process of protein synthesis, during myocardial hypoxia or ischemia, they can be metabolised to TCA cycle substrates [91,92].

Hakuno et al., using the profiling of AAs and their derivatives in plasma, identified the correlations of individual AA with heart function in patients with systolic HF. The plasma concentrations of 41 AAs were analysed, and it was found that 17 of them changed significantly in patients with HF, 15 of which were higher than in the control group. In addition, the results of the study indicate a relationship between higher circulating AA concentrations and deterioration in heart function [93]. It may be related to the intensification of the breakdown of skeletal muscles, which serve as a reservoir of AAs [12].

### 3.5. Mitochondria

Changes in mitochondrial metabolism are a hallmark of the failing heart [17]. Their role is not yet fully understood and some evidence regarding the basic mechanisms remains contradictory, but it is considered likely that the combination of disorders found in the mitochondria of the failing heart contributes to the pathogenesis and progression of HF [2]. There are a number of studies available confirming the presence of mitochondrial dysfunction and the reduction of mitochondrial energy production in HF [49,94,95,96]. It is worth emphasising that mitochondrial dysfunction occurring in HF includes disturbances in energy production (lowering the synthesis of ATP, phosphocreatine, and the phosphocreatine/ATP ratio), as well as altered mitochondrial Ca^2+^ handling and the increasing emission of reactive oxygen species (ROS) and other free radicals [9,97,98,99].

The mitochondrial dysfunction described in HF results from structural and functional changes and disturbances in their dynamics [17]. Structural changes include altered composition and organisation of the organelle membrane. Functional changes mainly concern the activity of the electron transport chain (ETC) and other processes related to energy metabolism. The dynamics disturbances are manifested in the deterioration of mitochondrial biogenesis, fission and fusion, and impaired mitophagy [100]. It has also been observed that the failing heart mitochondria have reduced matrix density and are swollen [17,101].

In the study in the rat overload-induced HF model, proteomic analysis revealed a decreased mitochondrial abundance of 6 of the 11 evaluated proteins participating in FAO [102]. These results are also confirmed in other works [77,103]. The available evidence points to the role of lipotoxicity in the causes of mitochondrial dysfunction. In a mouse model of lipotoxicity, it was shown that intracellular accumulation of FA led to abnormal mitochondrial morphology, disturbed mitochondrial respiration, and increased ROS emissions [9,104,105]. Moreover, downregulation of PGC1α activity was found in both patients and experimental HF models. PGC1α is under the control of intermediates of lipid metabolism and participates in the regulation of mitochondrial biogenesis, which may be another mechanism linking FA accumulation with mitochondrial dysfunction [9,104,106].

Another transcription factor potentially associated with mitochondrial dysfunction is NRF1. Its role has been confirmed as a positive regulator of cardiac oxidative metabolism and mitochondrial biogenesis, which is epigenetically interrupted by DNA methylation in HF [13]. Impairment of mitochondrial biogenesis is described as an early phenomenon in the pathophysiology of HF [2,107]. The reduction of mitochondrial deoxyribonucleic acid (mtDNA) copies and the number of mitochondria in cardiomyocytes of failure of the heart has been confirmed in both patient studies and experimental HF models [108]. Regeneration of mitochondria through biogenesis has been recognised as necessary to maintain their functionality [109].

The increase in ROS emissions appears to be both the effect and the cause of damage to the mitochondria in the failing heart. ROS is formed, also under physiological conditions, as a by-product of mitochondrial ATP production [2]. Oxidative stress in the course of HF increases the activity of oxidases, which are partly responsible for the overproduction of ROS [110]. Further factors such as hyperglycaemia, antioxidant deficiency, and disturbed oxidation processes are mentioned as contributing to the accumulation of ROS [2,111]. Excess ROS causes peroxidation of the mitochondrial membrane phospholipid cardiolipin, which results in the impairment of ETC, reduction of FAO oxidation, and promotion of cardiomyocyte apoptosis [112]. Additionally, mtDNA is particularly susceptible to damage by ROS, which results in mutations that are related, inter alia, to the lack of histones, which protect DNA against this type of damage [108]. PGC-1α plays a role in maintaining the mitochondrial antioxidant defence, which is known to decrease in HF, further exacerbating oxidative stress and mitochondrial damage [108,113]. Due to the above, and because mitochondria are the main source of ROS in the cell, they are the most susceptible to oxidative damage [108]. In addition, ROS has been shown to impair cellular structures such as excitation–contraction coupling proteins [114] and regulate numerous signalling cascades, e.g., associated with hypertrophy [108].

## 4. Sepsis-Induced Cardiomyopathy

Sepsis has been described as ‘life-threatening organ dysfunction caused by a dysregulated host response to infection’ and is considered the most common cause of death in critically ill patients [3,4,5]. One of the organs most frequently affected by sepsis dysfunction is the heart [6]. In the literature, heart dysfunction associated with sepsis appears under several names, including septic cardiomyopathy [6], sepsis-induced cardiomyopathy (SIC or SICM) [4,115], and sepsis-induced myocardial dysfunction (SIMD) [116]. There is no uniform definition and criteria for the diagnosis of this disease entity. In this paper, the broad definition proposed in 2018 by Martin et al., defining SICM as ‘a sepsis-associated acute syndrome of cardiac dysfunction unrelated to ischemia with one or more of the main characteristics: left ventricular dilatation with normal- or low-filling pressure, reduced ventricular contractility, and right ventricular dysfunction or left ventricular (systolic or diastolic) dysfunction with a reduced response to volume infusion’ [6]. However, it should be borne in mind that narrower criteria are also proposed, e.g., those presented by L’Heureux et al. [4]. Depending on the adopted criteria, the incidence of SICM in patients with sepsis is estimated at 10 to 70% and is associated with mortality rates of up to 70% [117,118]. It has been shown that the incidence of SICM increases with the severity of the disease, its occurrence is a significant factor contributing to organ dysfunction and is an unfavourable prognostic factor [6,115]. In addition to the features listed in the criteria, a minimal incidence of apoptosis and cardiomyocyte necrosis is characteristic of SICM [119]. Studies with autopsy and experimental animal models classify sepsis-induced changes in the heart as inflammatory cardiomyopathy [116].

Various mechanisms involved in the pathophysiology of SICM have been proposed, and it is assumed that it may be the result of their interaction [6,116]. Some of the mentioned mechanisms include the action of pro-inflammatory cytokines, pathogen-associated molecular patterns (PAMPs) and damage-associated molecular patterns (DAMPs), inflammation, metabolic disorders, excessive production of nitric oxide (NO), excessive production of ROS, decreased adrenergic response of cardiomyocytes, and changes in gene expression [6,116,120]. These factors may contribute to the disturbance of cardiomyocyte contractions by affecting intracellular calcium or the integrity of myofilaments [121,122]. 

It has been proposed that hypoxia, acidosis, hypovolaemia, and coagulation disorders contribute to the pathogenesis of SICM [123,124]. However, there is currently no evidence to support the hypothesis of global heart ischemia, once proposed as the underlying mechanism of SICM [125]. Cunnion et al. showed that patients with sepsis have preservation of coronary flow, the increased availability of oxygen, and the net myocardial lactate extraction [126]. Similar results were obtained by Dhainaut et al., who described preservation of the coronary flow and low oxygen extraction, which may indicate a defect in oxygen utilisation at the cellular level [55,127]. 

### 4.1. Mitochondrial Dysfunction in Sepsis-Induced Cardiomyopathy

Although there is limited and indirect evidence of cardiomyocyte mitochondrial dysfunction in sepsis in humans, there is evidence from experimental models for its involvement in the pathophysiology of SICM [115]. Since the cause–effect relationship has not been unequivocally established thus far, both the theory of the causal role of mitochondria in the pathogenesis of SIMC and the epiphenomena theory, which postulates the adaptive–protective role of changes in their functioning, are taken into account [115,121]. In discussing this issue, it is important to recognise the limited availability of myocardial tissue in sepsis patients; hence, the available evidence comes from autopsy materials and experimental models [128,129,130]. Moreover, in some studies in experimental models of sepsis, only its effect on cardiomyocytes has been assessed, without describing the clinical features of SICM [130,131]. In addition, some evidence of mitochondrial dysfunction in sepsis comes from studies using other, more accessible tissues such as skeletal muscle and blood cells [132,133]. Another limitation of research in SICM is the lack of consensus on the definition and criteria of this disorder [4,6,117].

**Figure 2:**
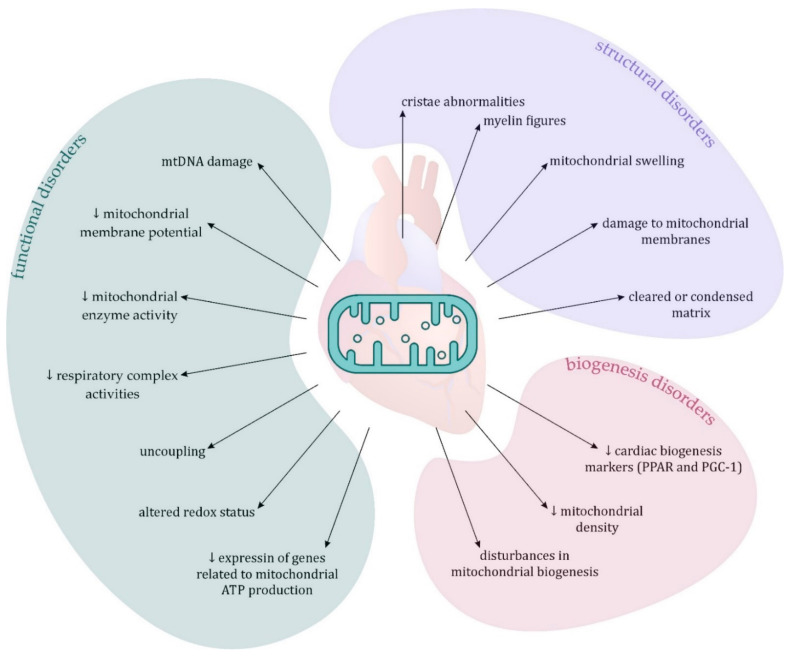
Changes in cardiomyocyte mitochondria in sepsis. Abbreviations: ATP = adenosine triphosphate, mtDNA = mitochondrial deoxyribonucleic acid; PGC-1 = peroxisome proliferator-activated receptor γ coactivator 1-α, PPAR = peroxisome proliferator-activated receptor, ↓ = decrease.

In studies using experimental models of sepsis, changes in mitochondria have been described, such as decreased ATP production and oxygen consumption, decreased mitochondrial enzyme activity, mtDNA damage, decreased mitochondrial membrane potential, and respiratory complex activities, as well as uncoupling and altered redox status [6,115,129,130]. Numerous structural changes in mitochondria have also been demonstrated, such as cristae abnormalities, swelling, cleared or condensed matrix, myelin figures, and damage to mitochondrial membranes [129,131,134,135]. In autopsy tissue studies, morphological changes in cardiomyocyte mitochondria were observed, such as mitochondrial swelling and damage to the cristae, with no irreversible cell damage (Figure 2). However, in this case, it cannot be ruled out that these changes are due to post-mortem deterioration [115,119]. There are also studies on experimental models in which, despite the presence of SICM and functional mitochondrial dysfunction, no structural changes in mitochondria have been demonstrated [136,137]. These data suggest that the presence of morphological abnormalities of mitochondria is not a prerequisite for their dysfunction and therefore do not allow for a clear assessment of their role in SICM [115].

Reduction of cardiac biogenesis markers such as PPAR and PGC-1 has been reported and has been shown to be associated with metabolic reprogramming, mitochondrial damage, and systolic dysfunction [6,115]. Disturbances in mitochondrial biogenesis, and consequently a decrease in mitochondrial density, are also postulated as a factor responsible for the reduction in mitochondrial respiratory activity observed in sepsis (Figure 2) [115,132]. Matkovich et al. compared the hearts of people who died from sepsis, the hearts of patients with ischemic or dilated cardiomyopathy who underwent heart transplantation, and non-failing hearts from brain-dead organ donors. It has been shown that compared to other cardiomyopathies, the hearts of patients who died from sepsis showed a marked decrease in the expression of genes related to mitochondrial ATP production [138]. The demonstration of this genetic reprogramming supports the theory that mitochondrial dysfunction in sepsis is related to functional rather than structural abnormalities. Moreover, these data suggest that the heart may respond to sepsis in a coordinated ‘programmatic’ fashion but do not provide a clear answer to the question of whether mitochondrial dysfunction in SICM is causal or epiphenomic.

Research also points to the role of mitophagy in cardiomyocytes during sepsis [139]. Organ dysfunction in the course of sepsis has been reported to be associated with impaired oxidative phosphorylation. The major mechanisms associated with the occurrence of oxidative phosphorylation disorders in SICM include the overproduction of ROS and NO, calcium overload, altered cAMP–PKA signalling, and depletion of intramitochondrial antioxidants [140,141]. Increased production of ROS and NO can cause direct damage (oxidative or nitrose), as well as inhibit oxidative phosphorylation complexes [140]. It has been shown that ROS and NO can inhibit mitochondrial complexes I and IV [142,143]. NO may contribute to mitochondrial dysfunction by increasing the permeability of their membranes [144]. In addition, increased activation of the mitochondrial inducible nitric oxide synthase (iNOS) is also responsible for increasing the mitochondrial levels of peroxynitrites, which have been shown to play a significant role in cardiomyocyte mitochondrial dysfunction during sepsis [140,145]. An experimental model study showed that mitochondrial dysfunction in the course of SICM is characterised by decreased rates of adenosine diphosphate (ADP) stimulated respiration (state 3 respiration) [146,147]. In addition, an increase in ADP-independent mitochondrial respiration (state 4 respiration) was found in cardiomyocytes in a mouse sepsis model, indicating uncoupling of oxidative phosphorylation [140,146]. In an experimental sepsis model, a reduction in the cardiac enzymatic activity of nicotinamide adenine dinucleotide (NADH) cytochrome c reductase, succinate cytochrome c reductase, and cytochrome c oxidase was demonstrated. The expression of proteins of mitochondrial complexes II and IV and the ATP content in cardiomyocytes also decreased [131]. Moreover, when assessing skeletal muscles, the correlation between the severity of septic shock and the decrease in the activity of the mitochondrial complex I, ATP depletion, and the decreased level of intracellular glutathione (acting as an antioxidant) was demonstrated [141].

The reduction of the mitochondrial membrane potential and ATP synthesis may also result from increased expression of mitochondrial uncoupling proteins (UCP) [148]. UCP induction separates the ATP synthesis process from oxygen consumption, which causes that some protons return to the matrix, bypassing F0F1. This lowers the ratio of ATP produced to oxygen consumed, reducing the efficiency of ATP production. On the other hand, UCP-induced proton leakage has the potential to reduce ROS formation [149]. The role of this phenomenon in the context of SICM is controversial, but it seems that it may have a protective effect [121,150]. 

Another suggested mechanism for the development of mitochondrial dysfunction related to oxidative and nitrative stress is the activation of poly (ADP-ribose) polymerase (PARP), an enzyme associated with numerous cellular processes, including DNA repair. Excessive activation of PARP may contribute to the formation of both inflammation and the development of metabolic disorders, by influencing the regulation of gene expression, impaired metabolism, and mechanisms leading to the production of alarmins (the role of PARP in sepsis is described in another study [151]). Although the primary location of PARP in the cell is the nucleus, it is known that their overactivation also impairs mitochondrial function [115]. Clinical trials have shown that there is significant PARP activation in the hearts of SICM patients and that the activity of PARP-1 in blood mononuclear cells was an independent risk factor for myocardial dysfunction in patients with septic shock [152,153]. Additionally, a study in an experimental model of sepsis has shown that pharmacological inhibition of PARP has a protective effect on the heart and is associated with an increase in ATP and nicotinamide adenine dinucleotide (NAD^+^) levels [154].

In patients with sepsis, altered levels of thyroid hormones—the so-called low T3 syndrome—have been known to affect mitochondrial function [155].

### 4.2. Changes in Substrate Utilisation in Sepsis-Induced Cardiomyopathy

**Figure 3:**
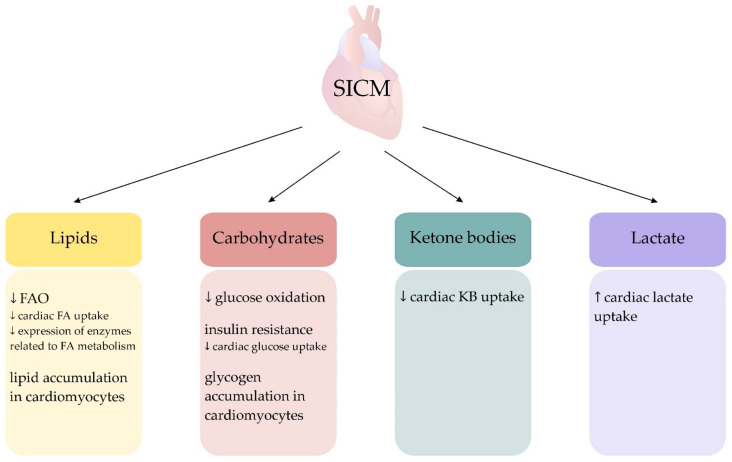
Changes in cardiac substrate metabolism in sepsis. Abbreviations: FA = fatty acids, FAO = fatty acid oxidation, KB = ketone bodies, ↓ = decrease, ↑ = increase.

Another characteristic disorder of septic hearts is a change in FA metabolism. In clinical sepsis studies, SICM has been shown to be accompanied by decreased cardiac FA uptake as well as lipid and glycogen accumulation in cardiomyocytes. These observations indicate a reduction in the share of FA in energy production (Figure 3) [55,156]. In a study by Rossi et al., the hearts of patients who died from sepsis were assessed and the accumulation of lipids inside cardiomyocytes was found, which may be the result of FAO disturbance and an imbalance between FAO and myocardial FA uptake [156]. These results have been confirmed by studies on experimental models of sepsis [157,158].

Numerous systemic metabolic disorders have been described in sepsis, and their review has been presented in other studies [159,160]. These disorders cause, inter alia, elevated levels of triglycerides and FFA in plasma, resulting from decreased lipid uptake by tissues and disturbances in intravascular lipolysis [161]. Decreasing FAO is common in HF and, as previously described, is usually accompanied by a compensatory increase in glucose utilisation [33]. SICM also reduces FAO, but due to insulin resistance and inhibition of glucose metabolism, glucose oxidation does not increase in the septic heart to compensate for the decreased FAO (Figure 1C) [54,55,162]. Chew et al., using microdialysis, assessed the level of metabolites such as glucose, lactate, and pyruvate in the myocardium in a porcine model of endotoxic and haemorrhagic shock. The study showed a rapid fall in myocardial glucose in endotoxic but not haemorrhagic shock. It has also been observed that in endotoxic shock there is an increase in the concentration of pyruvate and lactate in the myocardium and there is no evidence of anaerobic metabolism of the myocardium [126,163]. Standage et al., in a study in a mouse model of sepsis, reported inhibition of PDH activity in the heart [137]. They also showed molecular changes occurring during sepsis that can inhibit the oxidation of glucose—including an increase in pyruvate dehydrogenase kinase 2 (PDK2) and pyruvate dehydrogenase kinase 4 (PDK4) protein levels, and an increase in PDH phosphorylation which inhibits the entry of pyruvate into the TCA cycle [137]. An interesting phenomenon in the heart of sepsis patients is a net lactate extraction between arterial and coronary sinus blood, with a simultaneously reduced uptake of other energy substrates (FFA, glucose, KB) [55,126]. Reducing the role of glucose as an energy substrate seems to be characteristic of SICM. Completely shut down of glucose oxidation during ischaemia is also described in myocardial infarction, but in this case, there is a gradual reactivation of glucose oxidation during reperfusion (activation of AMPK by the ischaemia, Randle cycle), with the further possibility of achieving higher glucose oxidation than in normoxic conditions (activation of PDH by the mitochondrial Ca^2+^ overload) [164]. Interesting results were presented by Zheng et al., who, in a study on an experimental sepsis model, showed that sepsis enhances glycolytic metabolism, which plays a role in SICM and mortality. They also found that modulation of glycolytic metabolism by administration of 2-deoxy-D-glucose (2-DG), an inhibitor for hexokinase-2 which is the initial kinase for glycolysis, improves cardiac function and survival outcomes in a mice model of sepsis. These results indicate that enhanced glycolytic metabolism contributes to cardiac dysfunction in sepsis and that its modulation in the early phase of sepsis could be an appropriate approach for sepsis [118].

The use of FAs in the heart in sepsis can be inhibited at many stages—in experimental models of sepsis, it has been shown that lipopolysaccharide (LPS) reduces the expression of enzymes related to FA metabolism, including acyl–CoA synthetase and carnitine palmitoyl transferase-1 [165,166]. LPS has also been shown to worsen cardiac uptake of FAs, which may be associated with decreased expression of heart-specific, fatty-acid-binding proteins (FABPs), fatty acid transporter/CD36 (FAT/CD36), and very-low-density lipoprotein receptor (VLDL-R) [167,168]. It has been observed that inflammatory signalling mediated by the TLR can induce downregulation of FAO-related receptors and transcription factors: PPARα, PPARγ, and its coactivator PGC-1, thyroid receptors (TR), retinoid X α receptor (RxR) [137,157,158,162,166]. Interestingly, restoration of FAO in the myocardium has been shown to prevent cardiac dysfunction and mortality despite the development of cardiac inflammation [157].

Standage et al. conducted a study in a mouse model of caecal ligation puncture (CLP) induced sepsis and were the first to announce that in the early stage of sepsis, there may be an increase in FAO with an increase in heart function [137]. In addition, the study compared wild-type mice with PPARα deletion (PPARα -/-) mice and showed that in sepsis, PPARα -/- mice had a relative deficiency of nonlipid substrates and an excess of lipid substrates that they were unable to utilise, as indicated as the cause of their increased mortality. The authors of the study proposed that enhancement of FAO is a necessary part of the adaptation process of the myocardium in early sepsis [137]. The study also found that although PPARα messenger ribonucleic acid (mRNA) expression and its transcriptional targets in sepsis decreased, there was a transient increase 24 h after CLP before the decline in these proteins. This corresponds to the enhancement of FAO in early sepsis and indicates that regulation of energy substrate utilisation in the septic heart is also modulated by mechanisms other than transcription regulation [137,169].

## 5. Conclusions

Metabolic disorders are a typical feature of the failing heart, and their nature and severity depend on the aetiology and stage of HF. Despite the increasing number of scientific reports on this problem, the place of these changes in the pathogenesis of HF has not been clearly established. Several hypotheses are proposed, according to the first, changes in the heart are initially adaptive, and then maladaptive, and then contribute to the progression of failure. The next ones compare the failing heart to ‘engine flooded with fuel’, overloaded with an excessive amount of energy substrates, and to ‘engine out of fuel’, in which this organ is energetically starved [12,33]. Regardless of which hypothesis is correct, the presence of metabolic disorders in the failing heart is unquestionable, which makes the issue appear to be a promising therapeutic target and this requires further research.

A unique form of cardiac dysfunction is SICM. Understanding the nature of this disorder is especially important because sepsis is a serious epidemiological problem, and the heart is one of the most frequently dysfunctional organs in sepsis. Moreover, despite the efforts of scientists, the modern therapy of sepsis is based on antimicrobial treatment and the support of failing organs. Innovative solutions in the treatment of patients with sepsis constitute a great, and so far unmet, need for medicine; therefore, it seems justified to pay attention to metabolic disorders, which are an important component of the dysregulated host response to infection. The study of metabolic disorders in the hearts of patients with SICM is burdened with limitations related, inter alia, to the difficulty of obtaining material for research and the lack of uniform criteria for this disease entity. An additional variable potentially hindering the study of cardiac metabolism in SICM is the coexistence of septic shock, and in this context, the effect of the use of vasopressors and inotropic drugs on cardiac metabolism. Nevertheless, the number of available reports continues to increase, revealing a picture of serious metabolic disturbances in cardiomyocytes, which may be a promising treatment target in the future.

## Figures and Tables

**Table 1:** The energy efficiency of non-nitrogenous energy substrates [61,62].

Substrate	P/O Ratio ^a^	Energy Liberated [kcal/mol C_2_ Units]
Palmitate	2.33	298.0 ^b^
Glucose	2.58	223.6
Pyruvate	2.50	185.7
β-hydroxybutyrate	2.50	243.6

^a^ Number of molecules of ATP produced per atom of oxygen reduced by the mitochondrial ETC; ^b^ there is loss of ATP due to uncoupling proteins generating heat instead of ATP. Abbreviations: ATP = adenosine triphosphate, C_2_ units = two-carbon units, ETC = electron transport chain, kcal = kilocalorie.

## Data Availability

Not applicable.

## Abbreviations

2-DG	2-Deoxy-D-glucose
AA	Amino acids
Acetyl–CoA	Acetyl coenzyme A
ADP	Adenosine diphosphate
AMP	Adenosine monophosphate
AMPK	5’AMP-activated protein kinase
ATP	Adenosine triphosphate
βHB	Β-hydroxybutyrate
CLP	Caecal ligation puncture
DAMP	Damage-associated molecular patterns
ETC	Electron transport chain
ERRα	Oestrogen-like receptor α
FA	Fatty acids
FABP	Fatty acid binding proteins
FAO	Fatty acid oxidation
FAT	Fatty acid transporter
HF	Heart failure
iNOS	Inducible nitric oxide synthase
KB	Ketone bodies
LCAC	Long-chain acylcarnitine
LPS	Lipopolysaccharide
mRNA	Messenger ribonucleic acid
mtDNA	Mitochondrial deoxyribonucleic acid
NAD+/NADH	Nicotinamide adenine dinucleotide
NO	Nitric oxide
NRF	Nuclear receptor factor
PAMP	Pathogen-associated molecular patterns
PARP	Poly (ADP-ribose) polymerase
PDH	Pyruvate dehydrogenase
PDK	Pyruvate dehydrogenase kinase
PGC-1	Peroxisome proliferator-activated receptor γ coactivator 1
PPARα	Peroxisome proliferator-activated receptor α
ROS	Reactive oxygen species
RxR	Retinoid X α receptor
SERCA	Sarcoendoplasmic reticulum Ca^2+^-atpase
SICM/SIC	Sepsis-induced cardiomyopathy
SIMD	Sepsis-induced myocardial dysfunction
TCA	Tricarboxylic acid
Tfam	Mitochondrial transcription factor A
TLR	Toll-like receptor
TLS	Toxic lipid species
TR	Thyroid receptor
UCP	Uncoupling proteins
VLDL-R	Very-low-density lipoprotein receptor
